# Biological significance and therapeutic implication of resveratrol-inhibited Wnt, Notch and STAT3 signaling in cervical cancer cells

**DOI:** 10.18632/genesandcancer.15

**Published:** 2014-05

**Authors:** Peng Zhang, Hong Li, Bin Yang, Fan Yang, Lin-Lin Zhang, Qing-You Kong, Xiao-Yan Chen, Mo-Li Wu, Jia Liu

**Affiliations:** ^1^ Liaoning Laboratory of Cancer Genetics and Epigenetics and Department of Cell Biology, College of Basic Medical Sciences, Dalian Medical University, Dalian 116044, China; ^2^ Department of Gynaecological Surgery, Sheng-Jing Hospital, China Medical University, Shenyang 110031, China

**Keywords:** Cervical cancers, Resveratrol, Molecular target, Signal transduction pathways, STAT3 signaling

## Abstract

Cervical cancers/CCs are one of the commonest malignancies and the second leading cause of cancer-related death in women. Resveratrol inhibits CC cell growth but its molecular target(s) remains unclear. Since the signaling pathways mediated by STAT3, Notch1 and Wnt2 play beneficial roles in CC formation and progression, the effects of resveratrol on them in cervical adenocarcinoma (HeLa) and squamous cell carcinoma (SiHa) cells were analyzed. The biological significances of the above signaling for HeLa and SiHa cells were evaluated by treating the cells with STAT3, Wnt or Notch selective inhibitors. The frequencies of STAT3, Notch and Wnt activations in 68 cases of CC specimens and 38 non-cancerous cervical epithelia were examined by tissue microarray-based immunohistochemical staining. The results revealed that HeLa and SiHa cells treated by 100μM resveratrol showed extensive apoptosis, accompanied with suppression of STAT3, Notch and Wnt activations. Growth inhibition and apoptosis were found in HeLa and SiHa populations treated by AG490, a STAT3/JAK3 inhibitor but not the ones treated by Notch inhibitor L-685,458 or by Wnt inhibitor XAV-939. Immunohistochemical staining performed on the tissue microarrays showed that the frequencies of Notch1, Notch2, Hes1, Wnt2, Wnt5a and p-STAT3 detection as well as β-catenin nuclear translocation in CC samples were significantly higher than that of noncancerous group (p<0.01), while the expression rate of PIAS3 was remarkably low in cancer samples (p<0.01). Our results thus demonstrate that STAT3, Wnt and Notch signaling are frequently co-activated in human CC cells and specimens and resveratrol can concurrently inhibit those signaling activations and meanwhile lead cervical squamous cell carcinoma and adenocarcinoma cells to growth arrest and apoptosis. STAT3 signaling is more critical for CC cells and is the major target of resveratrol because selective inhibition of STAT3 rather than Wnt or Notch activation commits SiHa and HeLa cells to apoptosis.

## INTRODUCTION

Cervical cancers (CC) are one of the leading causes of cancer-related death among women in developing countries [1,2], which are classified into squamous cell carcinomas and adenocarcinomas according to their cellular origins [3]. Surgery is still the first choice of CC treatments, but frequent relapse and metastasis lead to poor prognosis of CC patients, especially those at advanced stage [4]. Chemotherapy has been widely used to prevent recurrence in postoperative management of CCs [5]. However, frequent drug resistance and severe toxicities damage patients' life quality [6]. It is therefore of clinical values to explore more reliable and less toxic therapeutic approach in the adjuvant treatment of cervical cancers.

Resveratrol (3, 5, 4′-trihydroxy-trans-stilbene), a phytoalexin, can be found in some edible food materials such as grape skins, pea-nuts and red wine [7,8]. A body of evidence shows that this compound has multiple biological activities including induction of differentiation and apoptosis of cancer cells [9,10]. For example, human medulloblastoma cells are sensitive to resveratrol in terms of growth arrest, neuron-oriented differentiation and distinct apoptosis [11]. And the growth of transplanted human transitional cell carcinomas in nude mouse urinary bladders can be efficiently suppressed by regular resveratrol installation [12]. More importantly, resveratrol has little harmful effect on glial cells and neurons in central nervous system and the tumor surrounding uro-epithelium [13,14], suggesting its potential values in the clinical treatments of those cancers. In the case of cervical cancers, resveratrol exerts radiosensitizing and anti-proliferative effects on them [15], but its underlying molecular mechanism remains to be investigated.

Resveratrol has multifaceted molecular effects on the treated cells. For instance, it can inhibit growth and induce apoptosis of human medulloblastoma and glioblastoma cells through suppressing the activations of several signaling pathways [16-18]. The current study thus aims to check 1) the statuses of STAT3-, Notch- and Wnt-mediated signaling in a squamous carcinoma cell line, SiHa, and an adenocarcinoma cell line, HeLa, of the cervix, 2) the influence of resveratrol in the biological activities of the three signaling pathways and 3) the biological consequence(s) of selective inhibition of individual signaling to the two cell lines.

## RESULTS

### Growth arrest and apoptosis of resveratrol-treated HeLa and SiHa cells

**Figure 1:**
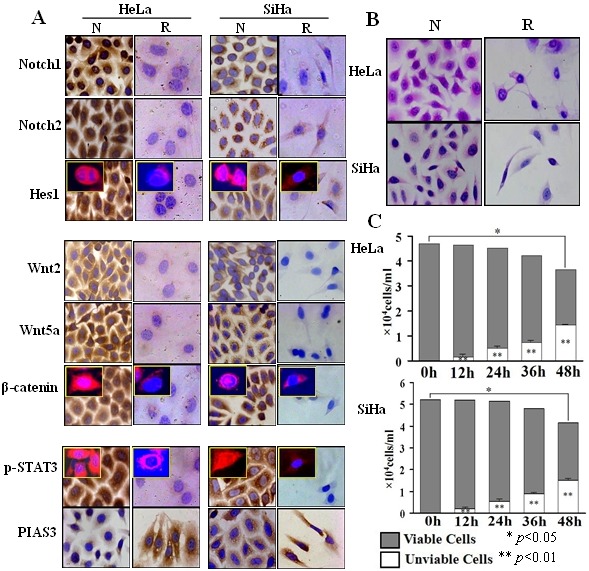
Inhibitory effects of resveratrol on cervical squamous cell carcinoma SiHa and adenocarcinoma HeLa cells A: Immunocytochemical illustration of intracellular distribution of Notch1, Notch2, Hes1, Wnt2, Wnt5a, β-catenin, p-STAT3 and PIAS3 in HeLa and SiHa cells without (N) and with (R) 100 μM resveratrol treatment for 48h. Immunofluorescent staining results were showed on the up-left corners. B: HE staining in HeLa and SiHa cells without (N) and with (R) 100 μM resveratrol treatment for 48h. C: Trypan blue discrimination of stained (unviable) and unstained (viable) cells. HeLa and SiHa cells were treated with 100 μM resveratrol for 0h, 12h, 24h, 36h and 48h respectively.

H/E morphologic staining demonstrated that HeLa and SiHa cells showed distinct apoptotic phenotypes after 100 μM resveratrol treatment for 48 hours (Figure 1B). Trypan blue cell discrimination assay revealed increased cell death fractions and significant cell number reduction (p<0.01; Figure 1C) in the two resveratrol-treated populations. Flow cytometry further demonstrated that the percentages of S phase and apoptotic HeLa cells were 34.14% and 0% under normal culture condition, which increased to 64.62% and 38.62% in resveratrol-treated population (Figure 3B: N and R; p<0.01). AnnexinV-FITC and PI double dye labelling showed that the apoptotic cells (on the low-right quadrant, FITC+/PI-) were 2% in normally cultured HeLa cells and reached to 39.1% after 48 hour 100 μM resveratrol treatment. The similar phenomena were also found in resveratrol-treated SiHa cells (Figure 3C).

### Resveratrol inhibited Notch, Wnt and STAT3 activation

**Figure 2:**
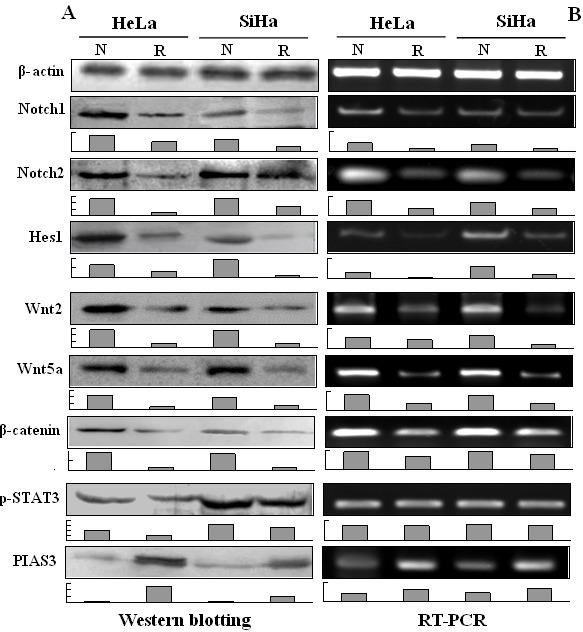
Statuses of STAT3, Notch and Wnt signaling in SiHa and HeLa cells and the influences of resveratrol in them A: Evaluation of Notch1, Notch2, Hes1, Wnt2, Wnt5a, β-catenin, p-STAT3 (STAT3) and PIAS3 in HeLa and SiHa cells without (N) and with (R) 100 μM resveratrol treatment for 48h by Western blotting and B: RT-PCR, β-actin was used as a quantitative control. Gray analyses of the Western blotting and RT-PCR results were under each picture, the ordinate was the ratio of each index and β-actin grey value.

Immunocytochemical/ICC staining (Figure 1A) showed that Notch1, Notch2, Wnt2 and Wnt5a were expressed in HeLa and SiHa cells and were downregulated when the cells were exposed to 100 μM resveratrol for 48 hours. Predominant nuclear labelling of Hes1, β-catenin and p-STAT3 were found in normally cultured HeLa and SiHa cells, which became weakened by resveratrol. The protein inhibitor of activated STAT3 (PIAS3) was expressed in low level in HeLa and SiHa cells under normal culture condition and was up-regulated after resveratrol treatment (Figure 1A). Paralleled RT-PCR and Western blot analyses showed that Notch1, Notch2, Hes1, Wnt2, Wnt5a and β-catenin were obviously down-regulated and PIAS3 was up-regulated in resveratrol-treated HeLa and SiHa cells (Figure 2A and 2B; p<0.05). Although the levels of STAT3 expression remained largely unchanged between normally cultured and resveratrol-treated HeLa or SiHa cells (Figure 2B), p-STAT3 levels were reduced in resveratrol-treated HeLa and SiHa cells in the extents of 51.8% and 19.7%, respectively (Figure 2A).

### Variable sensitivities to Notch, Wnt and STAT3 inhibitors

**Figure 3:**
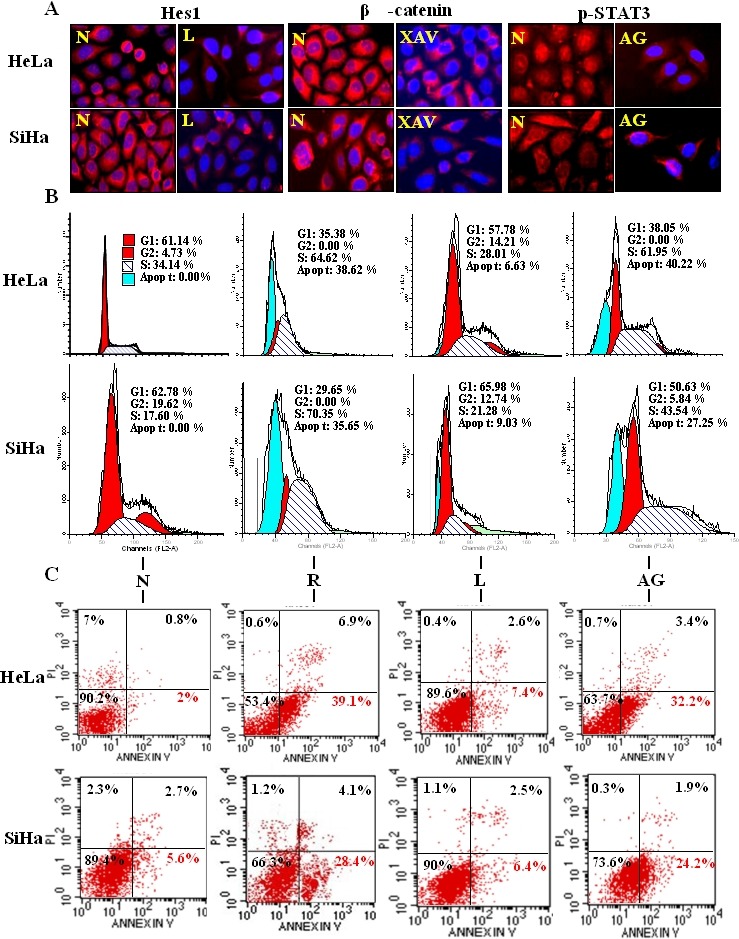
Evaluation of the effects of STAT3, Notch and Wnt selective inhibitors on HeLa and SiHa cells A: Immunofluorescent staining of Hes1 in HeLa and SiHa cells without (N) and with (L) 8 µM L685,458 treatment for 48h. Immunofluorescent staining of β-catenin in HeLa and SiHa cells without (N) and with (XAV) 10 μM XAV-939 treatment for 48h. Immunofluorescent staining of p-STAT3 in HeLa and SiHa cells without (N) and with (AG) 80 μM AG490 treatment for 48h. B: Flow cytometry analyses of cell cycle fractions in HeLa and SiHa cells. N: normal cultured cells, R: 100 μM resveratrol treated for 48h, L: 8 µM L685,458 treated for 48h, AG: 80 μM AG490 treated for 48h. C: Flow cytometry analyses of cell apoptosis fractions in HeLa and SiHa cells. PI/AnnexinV-FITC double staining. The percentage of each group of data was in the quadrant, UR: necrotic cells (FITC-/PI-), LL: viable cells (FITC+/PI+), LR: apoptotic cells (FITC+/PI-).

To evaluate potential biological significance of Notch, Wnt and STAT3 activations in the two CC cells, the selective inhibitors of the three signalling pathways were used to treat HeLa and SiHa cells, respectively. The results revealed that 8 μM L-685,458 blocked Notch activation in the two cell lines in terms of reduced cytoplasmic distribution and almost diminished nuclear labelling of Hes1 proteins (Figure 3A). However, this treatment neither brought about growth arrest and cell death nor the cell cycle alteration. (Figure 3B and 3C: N and L). XAV-939-treated HeLa and SiHa cells showed reduction of cytoplasmic distribution and nuclear translocation of β-catenin without distinct morphologic change and growth inhibition (Figure 3A). STAT3 phosphorylation was inhibited in HeLa and SiHa cells upon 80μM AG490 treatment for 48 hours (Figure 3A). 61.95% of AG490-treated HeLa cells were arrested in S-phase and 40.22% underwent apoptosis. In the case of AG490-treated SiHa cells, the S-phase and apoptotic cells were 43.54% and 27.25% respectively in comparison with 17.60% and 0% of their normally cultured counterparts (Figure 3B; p<0.05).

### Frequent co-activations of Notch, Wnt and STAT3 signaling in cervical cancer specimens

**Figure 4:**
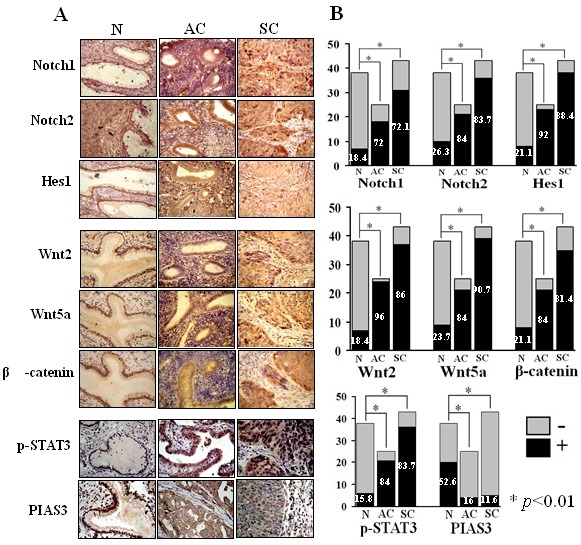
Frequencies of Notch1, Notch2, Wnt2 and Wnt5a detection and nuclear translocation of Hes1, β-catenin, p-STAT3 and PIAS3 in human cervical cancer specimens A: Tissue microarray-based immunohistochemical staining for Notch1, Notch2, Hes1, Wnt2, Wnt5a, β-catenin, p-STAT3 and PIAS3 in normal cervical tissues removed from the uterine fibroid patients at post-reproduction ages (N), cervical adenocarcinomas (AC) and cervical squamous cell carcinomas (SC) (original magnifications×400). B: Histogram of Table 1, *p<0.01.

The results of tissue microarray-based immunohistochemical staining were summarized in Table 1 and shown in Figure 4. It was found that p-STAT3 could be detected in 6 of 38 (15.8%) non-cancerous cervical epithelia, in 84.0% (21/25; p<0.01) adenocarcinomas and in 36 of 43 (83.7%; p<0.01) squamous cell carcinomas. Similarly, the frequencies of Notch 1, Notch2, Wnt2 and Wnt5a detection as well as nuclear translocation of Hes1 and β-catenin in CCs were significantly higher than the noncancerous group (p<0.01). The expression rates of intrinsic STAT3 inhibitor PIAS3 were 52.6% (20/38) among noncancerous epithelial tissues but reduced to 16.0% (4/25; p<0.01) and 11.6% (5/43; p<0.01) in cervical adenocarcinoma and squamous carcinoma samples, respectively.

**Table 1:** Immunohistochemical profiling of Notch1, Notch2, Hes1, Wnt2, Wnt5a, β-catenin, p-STAT3 and PIAS3 expression in human cervical tissues (N: normal cervical tissues removed from the uterine fibroidpatients at post-reproduction ages, AC: cervical adenocarcinomas, SC: cervical squamous cell carcinomas. * p<0.01)

	N			AC				SC			
	−	+	≥++	−	+	≥++	p	−	+	≥++	p
Notch1	31	6	1	7	10	8	*	12	20	11	*
Notch2	28	5	5	4	16	5	*	7	15	21	*
Hes1	30	3	5	2	18	5	*	5	19	19	*
Wnt2	31	4	3	1	14	10	*	6	17	20	*
Wnt5a	29	2	7	4	13	8	*	4	15	24	*
β-catenin	30	8	0	4	10	11	*	8	17	18	*
p-STAT3	32	6	0	4	17	4	*	7	19	17	*
PIAS3	18	20	0	21	3	1	*	38	4	1	*

## DISCUSSION

Cervical carcinoma is the second commonest malignant tumor of women in the world [19]. The outcome of localized cervical cancers treated by surgery and/or chemo-radiation therapies is promising, while the prognosis of Stage III and IV cervical cancers is poor with 5-year survival rates of 43.2% and 13% respectively, because of the extensive local invasion and frequent distal metastasis [20-22]. Cisplatin and paclitaxel are currently used as the first-line chemotherapeutic drug for cervical cancers but its efficacy is not promising because of the drug resistance and the serious adverse reactions [23,24]. It is therefore in urgent need to explore a safer and more effective agent against cervical cancers [25,26].

Resveratrol is increasingly recognized as a potential cancer preventive and therapeutic agent [27]. A body of evidence indicates that resveratrol has inhibitory effects on many types of malignancies including the squamous cell carcinomas and adenocarcinomas of uterus cervical cancers [28,29]. Nevertheless, the molecular mechanism underlying the effects of resveratrol on cervical cancers remains unclear. On the other hand, multiple cancer associated signaling pathways are activated in human cervical cancers and considered as potential therapeutic target(s) in the treatment of those cancers [30-33]. Notch signaling in CD66^+^ cells drives the progression of human cervical cancers [34]. The in vivo growth, invasion and angiogenesis of cervical cancer can be inhibited by Wnt inhibitory factor 1 [35]. And STAT3 signaling is constitutively activated in cervical cancer and may play potential role in progression of HPV16-mediated cervical carcinogenesis [36]. A body of evidence demonstrates that the multifaceted biological activities of resveratrol are reflected by its inhibitory effects on the activations of cancer-associated signaling pathways [37-39]. For these reasons, the statuses of Wnt, Notch and STAT3 signaling in HeLa and SiHa cells without and with resveratrol treatment were investigated in currently.

It was found that resveratrol in the dose of 100μM efficiently inhibited the growth and suppressed Wnt2, Wnt5a, Notch1 and Notch2 expression of the two cell lines. Although transcriptional levels of STAT3 remained almost unchanged between the normally cultured and resveratrol-treated HeLa and SiHa cells, phosphorylation of STAT3 was obviously inhibited by resveratrol, accompanied with distinct PIAS3 up-regulation, indicating that these two events may be the main reasons of STAT3 inactivation. These findings further confirm the multiple targeting features of resveratrol and, meanwhile, suggest potential therapeutic effectiveness of resveratrol on both squamous cell carcinomas and adenocarcinomas of human cervical uterus. Given the reduced activities of Wnt, Notch and STAT3 signaling in resveratrol-treated cervical cancer cells, it would be worthwhile to evaluate the significance of Wnt, Notch or STAT3 signaling for CC cells and the biological consequence of their inhibition.

One of the efficient approaches to block the pathway of signal transduction is to employ an appropriate selective inhibitor [40,41]. Therefore, XAV-939, L-685,458 and AG490 were used to respectively inhibit Wnt-, Notch- and STAT3-mediated signaling in HeLa and SiHa cells. Although nuclear translocation of β-catenin was remarkably reduced by XAV-939, HeLa and SiHa cells with the treatment showed little morphological change and growth arrest, suggesting that Wnt signaling might not be crucial for the two types of cervical cancer cell lines. Hes1 is the downstream gene of Notch signaling [42]. In L-685,458-treated cells, Hes1 nuclear translocation was largely blocked and about 6.63% of HeLa and 9.03% of SiHa cells were subjected to apoptosis, indicating that Notch signaling may play certain favourable roles in the two cell lines. When AG490 was used, STAT3 phosphorylation and nuclear translocation in HeLa and SiHa cells were successfully inhibited. More importantly, S-phase arrest was found in AG490-treated SiHa (43.54%) and especially HeLa cells (61.95%), accompanied with remarkable apoptosis (27.25% and 40.22%). These phenomena are very similar with that of resveratrol-treated cells in which the fractions of S-phase and apoptosis increase to 70.35% and 35.65% in SiHa and to 64.62% and 38.62% in HeLa cells. It is therefore strongly suggested that among the three resveratrol inhibited signaling pathways so far checked, inactivation of STAT3 signaling is the lethal molecular event in Hela and SiHa cells. Alternatively, the activated STAT3 signaling seems more crucial than the other two in the proliferation and survival of human cervical cancers although Notch signaling may also play certain roles in those processes. In this context, STAT3 targeting strategy would be applicable in the management of this sort of woman malignancies and the less toxic resveratrol with multifaceted actions is an optimal choice.

The involvements of Wnt, Notch and STAT3 signaling in cervical carcinogenesis have been described [43-45]. However, the paralleled analyses of their statuses in a panel of CC and noncancerous specimens are still limited. Since activated Notch and, especially STAT3 signaling as the molecular targets of resveratrol are critical in the growth and survival of SiHa and HeLa cells, the frequencies of Wnt, Notch and STAT3 activation in the two types of CCs were elucidated to correlate the in vitro findings with the in vivo statuses. Our immunohistochemical results revealed that in comparison with the data obtained from the noncancerous cervical mucosa, these three signaling pathways are frequently activated (p<0.01) and the expression of PIAS3 was uncommon (11.6%, 5/43, p<0.01) in cervical cancers irrespective to their cell origins. In accordance to the findings from SiHa and HeLa cells, Wnt, Notch and STAT3 co-activations could be detected in 79.1% (34/43) of squmous cell carcinomas and 80% (20/25) of adenocarcinomas, suggesting their potential crosstalk in regulating cancer-related gene expression during the stepwise cervical carcinogenisis [46,47]. Since resveratrol can concurrently inactivates Wnt, Notch and STAT3 signaling in SiHa and HeLa cells, this multi-targeting compound would be of practical values in the prevention and treatment of cervical squamous cell carcinomas and adenocarcinomas.

## MATERIALS AND METHODS

### Cell culture and treatments

Human cervical adenocarcinoma cell line HeLa (Cat No: TCHu187) and human cervical squamous carcinoma cell line SiHa (Cat No: TCHu113) were provided by Cell Bank of Academia Sinica, Shanghai. HeLa cells were cultured in Roswell Park Memorial Institute 1640 Medium (RPMI1640, Gibco Life Science, Grand Island, NY, USA) and SiHa cells were cultured in MEM essential medium containing 10% fetal bovine serum (Gibco Life Science, Grand Island, NY, USA) under 37°C and 5% CO_2_ condition. They (5×10^4^/ml) were plated to Ø 100 mm dishes (NUNC, Denmark) and incubated for 24h before further experiments. Resveratrol (Sigma Chemical, Inc, St. Louis, MO) was dissolved in dimethylsulfoxide (DMSO, Sigma) to a stock concentration of 100mM and diluted with culture medium to an optimal working concentration of 100μM just before use [48]. For H/E morphologic and immunocytochemical/ICC staining, the coverslips were put into the dishes before initial cell seeding and collected regularly during the experiments.

### Morphologic examination and cell proliferation assay

Total cell numbers and cell viability of HeLa and SiHa cells without and with 100μM resveratrol treatment were determined in 12-h intervals by staining the single cell suspensions with 2% trypan blue and counting the stained and unstained cells with the haemocytometers. The effects of resveratrol and the selective inhibitors of Wnt (8 µM L685, 458), Notch (10μM XAV-939) and STAT3 (80μM AG490) pathway on cell cycle and apoptosis of HeLa and SiHa cells were evaluated by flow cytometry. For staining with DNA dye, the cells were suspended in 0.5 ml to 1 ml of propidium iodide solution containing RNase and incubated at 37°C for 30 minutes. Cell cycle and cell apoptosis profiles were obtained with a FACSvantage flow cytometer (Becton Dickinson, San Jose, CA). The data obtained were analyzed with ModFit software (Verity Software House, Inc, Topsham, ME).

### Immunocytochemical and immunofluorescent staining

Immunocytochemical staining (ICC) was performed on the coverslips obtained from each of the experimental groups. The antibodies used were a mouse anti-human Notch1 monoclonal antibody (1:100, NeoMarkers, Inc, Fremont, California), a rabbit anti-human Notch2 polyclonal antibody (1:100, Santa Cruz, CA, USA), a rabbit anti-human Hes1 polyclonal antibody (1:1000; a generous gift of Tetsuo Sudo, PhD, Toray Industries, Tokyo, Japan), a goat anti-human Wnt2 (1:100; Santa Cruz, CA, USA), a rabbit anti-human Wnt5a polyclonal antibody(1:100; Santa Cruz, CA, USA), a mouse anti-human β-catenin (1:100; Santa Cruz, CA, USA), a rabbit anti-human p-STAT3(1:100; Santa Cruz, CA, USA) and a rabbit anti-human PIAS3 polyclonal antibody (1:100; Santa Cruz, CA, USA). All of the antibodies were used according to the manufacturer's instruction. ICC staining was conducted by the method described elsewhere [16]. For immunofluorescent (IF) staining, the coverslips were rinsed with PBS (pH 7.4), fixed for 20 minutes in 80% cold acetone and stored at −20°C until use. After being blocked with 10% goat serum in PBS for 20 minutes, the coverslips were incubated overnight with primary antibodies against Hes1, β-catenin and p-STAT3 in humid chamber at 4°C, followed by co-incubation with fluorescence-labeled goat anti-rabbit and rabbit anti-mouse IgG (1:100; Santa Cruz, CA, USA) in a 37°C humid chamber for 60 minutes in darkness. DAPI (4',6-diamidino-2-phenylindole,2-(4-amidinophenyl)-1H-indole-6-carboxamidine) was used to stain nuclei with blue fluorescent. After being sealed with 90% glycerol, the coverslips were observed and photographed (DP70 Digital Camera; Olympus, Tokyo, Japan) under a fluorescence microscope (BX51; Olympus).

### RNA isolation and reverse transcription-polymerase chain reaction

Total cellular RNA was isolated from each of experimental groups using Trizol solution (Life Technologies, Grand Island, NY). Reverse transcription (RT) was performed on RNA samples, followed by polymerase chain reaction (PCR) amplification. For RT, 0.5 μg of the RNA sample was added to 20μl of RT reaction mixture (Takara, Inc, Ltd, Dalian, China) containing 4μl of MgCl_2_, 2μl of 10 × RNA PCR buffer, 9.5μl of RNase-free distilled H_2_O, 2μl of deoxyribonucleotide triphosphate mixture, 0.5μl of RNase inhibitor, 1 μl of AMV reverse transcriptase, and 1μl of oligo dT-adaptor primer. The reaction was carried out by treating the samples at 55°C for 30 minutes, at 99°C for 5 minutes, and at 5°C for 5 minutes. Polymerase chain reaction was conducted using the primers specific for each of the target genes (Table 2). Briefly, 2.5μl of RT products were mixed with 16μl of PCR-grade water, then with 6.5 μl of PCR working solution containing 1×PCR buffer, 1μl of deoxyribonucleotide triphosphate, 2.5 units of Taq DNA polymerase, and 50 pM upstream and downstream primers for human Notch1, Notch2, Hes1, Wnt2, Wnt5a, β-catenin, STAT3 and PIAS3 respectively. Polymerase chain reactions for individual genes were performed according to the conditions reported elsewhere [49-57]. The PCR products were resolved on 1% agarose gel containing ethidium bromide (0.5μg/ml). The bands were visualized and photographed using UVP Biospectrum Imaging System (UVP, Inc, Upland, CA). The PCR products generated from the same RT solution by a pair of β-actin primers were cited as internal quantitative controls.

**Table 2:** The primer sequences of polymerase chain reactions

Parameters	Primer Sequence (5'→3')	Product size	Reference
β-actin	f: 5' - GCA TGG AGT CCT GTG GCA T - 3'	326	49
	r: 5' - CAT GAA GCA TTT GCG GTG G - 3'		
Notch1	f: 5' - TGT GAC AGC CAG TGC AAC TC - 3'	577	50
	r: 5' - TGG CAC TCT GGA AGC ACT GC - 3'		
Notch2	f: 5' - AAT GTC ATG GCC GCT TCA GAG - 3'	533	51
	r: 5' - TCG TGC AAG AGC CAG TTA CCC - 3'		
Hes1	f: 5' - CCA GTT TGC TTT CCT CAT TCC - 3'	240	52
	r: 5' - TCT TCT CTC CCA GTA TTC AAG TTC C - 3'		
Wnt2	f: 5' - GCC ACA CGC TGC ACC TAA AGC - 3'	379	53
	r: 5' - CAA TTA CCC TAA GGG TGG TAG C - 3'		
Wnt5a	f: 5' - CTA ACT TAG CTG TGT GGG ACA TG - 3'	254	54
	r: 5' - AAA TGC AGA AAG CAA GCT AGC AG - 3'		
β-catenin	f: 5' - ACA AAC TGT TTT GAA AAT GG - 3'	298	55
	r: 5' - CGA GTC ATT GCA TAC TGT CC -3'		
STAT3	f: 5'- GGG TGG AGA AGG ACA TCA GCG GTA A - 3'	198	56
	r: 5' - GCC GAC AAT ACT TTC CGA ATG C - 3'		
PIAS3	f: 5' - ACG CTG TTG GCC CCT GGC AC - 3'	322	57
	r: 5' - GGG GCT CGG CCC CAT TCT TGG - 3'		

### Protein preparation and Western blotting

Total cellular proteins were prepared from the cells under different culture conditions by the method described previously [16]. For Western blot analyses, the sample proteins (50μg/well) were separated by electrophoresis in 10% sodium dodecylsulfate-polyacrylamide gel electrophoresis (SDS-PAGE), transferred to polyvinylidene difluoride membrane (Amersham, Buckinghamshire, UK). The membranes were blocked with 5% skimmed milk in TBS-T (10 mM Tris-Cl, pH 8.0, 150 mM NaCl, and 0.5% Tween 20) at 4°C overnight, rinsed three times (10 minutes each time) with TBS-T, followed by 3 hours of incubation at room temperature with the first antibodies in appropriate concentrations (Notch1: 1:800, Notch2: 1:800, Hes1: 1:2500, Wnt2: 1:500, Wnt5a: 1:500, β-catenin:1:400, p-STAT3: 1:800, PIAS3: 1:1000), followed by 1 hour incubation with HRP-conjugated anti-mouse or -rabbit IgG (ZymedLab, Inc). The bound antibody was detected using the enhanced chemiluminescence system (Roche GmbH, Mannheim, Germany). After removing the labelling signal by incubation with stripping buffer (62.5mM Tris-HCl, pH 6.7, 100 mM 2-mercaptoethanol, 2% SDS) at 55°C for 30 minutes, the membrane was reprobed with other antibodies one by one by the same experimental procedures until all of the parameters were examined.

### Inhibition of Wnt, Notch and STAT3 signaling with selective inhibitors

L-685,458 (Calbiochem, San Diego, CA) is a potent and selective γ-secretase inhibitor, which inhibits Notch activation [58]. XAV-939 (Selleck, Houston, Texas, USA) selectively suppresses the transcription of Wnt/β-catenin through inhibiting tankyrase1/2 [59]. AG490 (Sigma, Inc, St. Louis, MO), a JAK-specific inhibitor, can suppress STAT3 signaling by inhibiting Tyr705 phosphorylation of STAT3 protein [60]. To evaluate the importance of the three signalling pathways in the growth and survival of CC cells, 8 μM L-685,458 [58], HeLa and SiHa cells were treated by 10 μM XAV-939 [59] and 80 μM AG490 [13], respectively. The treatments lasted for 72 hours and the cells were observed in 12 hour intervals. The cell bearing coverslips prepared were subjected to further analyses. The experiments were repeated for 3 times to establish confidential conclusion.

### Tissue microarray-based immunohistochemical staining

The archived paraffin blocks of 25 cases of adenocarcinomas, 43 squamous cell carcinomas cervical cancers and 38 normal cervical tissues removed from the uterine fibroid patients at post-reproduction ages. Tissue microarrays in the densities of 56 spots/cm^2^ were constructed by the method described elsewhere [33]. The microarrays prepared were sectioned to 5-μm thickness for immunohistochemical profiling of Notch1, Notch2, Hes1, Wnt2, Wnt5a, β-catenin, p-STAT3 and PIAS3 expression and intracellular distribution, respectively. The antibodies used were the same as that used in immunocytochemistry. The tissue microarray sections lacking incubation with individual primary antibodies were used as background controls. The staining results were evaluated separately by two investigators, with the intensity of immunolabeling scored as negative (−), weakly positive (+), moderately positive (++) or strongly positive (+++).

### Statistical analysis

The results of cell counting were evaluated with the independent-samples t-test and ANOVA. Mann-Whitney tests were used to analyze the expression status in different histological groups. Statistical significance was defined as p<0.05.
